# Detoxification of a pyrolytic aqueous condensate from wheat straw for utilization as substrate in *Aspergillus oryzae* DSM 1863 cultivations

**DOI:** 10.1186/s13068-022-02115-z

**Published:** 2022-02-17

**Authors:** Christin Kubisch, Katrin Ochsenreither

**Affiliations:** Institute of Process Engineering in Life Sciences 2-Technical Biology, Karlsruhe Institute of Technology (KIT), Fritz-Haber-Weg 4, 76131 Karlsruhe, Germany

**Keywords:** Fast pyrolysis, Pretreatment, Fungal biotechnology, Lignocellulose valorization, Acetate utilization

## Abstract

**Background:**

The pyrolytic aqueous condensate (PAC) formed during the fast pyrolysis of wheat straw contains a variety of organic carbons and might therefore potentially serve as an inexpensive substrate for microbial growth. One of its main components is acetic acid, which was recently shown to be a suitable carbon source for the filamentous fungus *Aspergillus oryzae*. However, the condensate also contains numerous toxic compounds that inhibit fungal growth and result in a tolerance of only about 1%. Therefore, to enable the use of the PAC as sole substrate for *A. oryzae* cultivations, a pretreatment seems to be necessary.

**Results:**

Various conditions for treatments with activated carbon, overliming, rotary evaporation and laccase were evaluated regarding fungal growth and the content of inhibitory model substances. Whereas the first three methods considerably increased the fungal tolerance to up to 1.625%, 12.5% and 30%, respectively, the enzymatic treatment did not result in any improvement. The optimum carbon load for the treatment with activated carbon was identified to be 10% (w/v) and overliming should ideally be performed at 100 °C and an initial pH of 12. The best detoxification results were achieved with rotary evaporation at 200 mbar as a complete removal of guaiacol and a strong reduction in the concentration of acetol, furfural, 2-cyclopenten-1-one and phenol by 84.9%, 95.4%, 97.7% and 86.2%, respectively, were observed*.* Subsequently, all possible combinations of the effective single methods were performed and rotary evaporation followed by overliming and activated carbon treatment proved to be most efficient as it enabled growth in 100% PAC shake-flask cultures and resulted in a maximum cell dry weight of 5.21 ± 0.46 g/L.

**Conclusion:**

This study provides a comprehensive insight into the detoxification efficiency of a variety of treatment methods at multiple conditions. It was revealed that with a suitable combination of these methods, PAC toxicity can be reduced to such an extent that growth on pure condensate is possible. This can be considered as a first important step towards a microbial valorization of the pyrolytic side-stream with *A. oryzae.*

**Supplementary Information:**

The online version contains supplementary material available at 10.1186/s13068-022-02115-z.

## Background

The need to cover the energy demand of mankind in times of a growing world population and emerging environmental concerns have raised the interest in more sustainable technologies for the production of fuels and bulk chemicals. A potential way to increase the sustainability of such production processes and to reduce their dependence on fossil-resources is the utilization of residual lignocellulosic biomass, as it represents a renewable feedstock that does not compete with food or feed production. However, lignocellulosic biomass is characterized by a rather complex and recalcitrant composition [1]. Therefore, a depolymerization of the lignocellulose structures is usually a prerequisite for its conversion to platform chemicals and fuels.

Fast pyrolysis is a thermochemical biomass conversion procedure and represents the first step of the bioliq^®^ process that was developed at the KIT (Karlsruhe Institute of Technology, Germany). During this process, shredded biomass is mixed with hot sand and is thereby heated up to temperatures of about 500 °C in the absence of oxygen [2]. Within 2–3 s the solid lignocellulosic feedstock is decomposed into char and vapors that are partly condensed to form a complex organic liquid, referred to as “bio-oil”, and non-condensable pyrolysis gases (for a more detailed description, see e.g. [2–4]). The utilization of ash-rich feedstocks like cereal straws and grasses [5] might cause a spontaneous or delayed phase separation of the bio-oil [6–8] that can be controlled by the implementation of a two-staged condensation as performed at KIT resulting in the separated formation of the organic bio-oil and an aqueous condensate [2]. The organic fraction is characterized by a high heating value of > 20 MJ/kg [9] enabling its gasification to produce syngas and after further processing synthetic fuels and chemicals. However, it contains only 50–70% of the initial bioenergy of the feedstock and is therefore mixed with up to 20% of the pyrolysis char to obtain a dense bio-slurry that makes about 90% of the bioenergy accessible for syngas production [3]. In contrast, the heating value of the PAC is rather low (5.4 MJ/kg) mainly due to its high water content of about 80–85% [2] making an energetic utilization rather unlikely.

Nevertheless, it still contains a variety of valuable organic compounds like organic acids, alcohols, aldehydes, ketones and phenolics (Additional file 1: Table S1) that can potentially serve as substrates for microbial fermentations. Current research approaches dealing with a microbial valorization of aqueous pyrolysis fractions range from the production of value-added compounds like lipids [10] and solvents [11–13] to their conversion into biogas via anaerobic digestion [14–16] and the utilization as substrate for H_2_ production in microbial electrolysis cells [17, 18]. However, the broad spectrum of carbons might also be detrimental for a potential microbial utilization of the condensate as it includes several compounds that could act as inhibitors for growth and production of various organisms [19, 20]. A multitude of procedures have already been described for the elimination of potential inhibitors from aqueous pyrolysis products and hydrolysates. Among them, the most common ones are extractions [11, 21], adsorption processes using activated carbon [22–25] or other adsorbents [26], ion exchange [27] as well as overliming [28–30] and rotary evaporation [10]. In addition to these physical and chemical methods, biological treatments using whole cells [31, 32] or enzymes [33] can also be applied to reduce the toxicity of liquid pyrolysis products. Especially peroxidases and laccases originating from lignin-degrading white rod fungi like *Trametes versicolor* were shown to efficiently remove phenolic substances [34, 35].

In many of the studies dealing with the microbial valorization of pyrolysis condensates and hydrolysates, the liquid substrate is not only treated by one, but a combination of several of the aforementioned detoxification methods [10, 26, 36–38]. However, the optimum order in which these methods should be performed to obtain the best detoxification result is usually not determined. Furthermore, many of them contain additional treatment steps like enzymatic or acid hydrolysis, which aim at the degradation of contained anhydrosugars into their monosaccharides to increase the fermentability of the aqueous substrate [11, 13]. However, as can be seen in Additional file 1: Table S1, the condensate produced at KIT does not contain sugar molecules, but mainly acetol and acetate, which should therefore be considered as the most promising substrates for a fermentative valorization of the pyrolytic side-stream. While publications dealing with microbial acetol conversion are scarce [12], many microorganisms are known to consume acetate as sole carbon source [10, 39–41]. For instance, it has recently been shown that the filamentous fungus *A. oryzae* is able to utilize high acetate concentrations of up to 70 g/L for its growth and l-malic acid production [42]. Moreover, the fungus is characterized by a high-tolerance against pyrolysis oils and also the impact of various PAC components on its growth and production behavior has been reported [19]. All these factors render *A. oryzae* a promising candidate for the microbial valorization of the unexploited side-stream. Therefore, the ascomycete was chosen in this work to first evaluate the potential of common detoxification methods for a further improvement of the fungal tolerance towards the aqueous condensate. The most effective methods were then selected for comprehensive combination experiments to determine the optimum detoxification procedure that allows the condensate to be utilized as sole substrate for growth of *A. oryzae.*

## Results

In order to enable the most effective detoxification of the PAC, the optimal conditions for a sole treatment with laccase (enzyme concentrations: 0–25 U/mL and durations: 0–24 h), activated carbon (carbon loads: 0–10% (w/v)), overliming (temperatures: 20–100 °C, different hydroxides: NaOH, Ca(OH)_2_ and pH strategies: initial pH 10, initial pH 12, adjustment to pH 10) or rotary evaporation (pressures: 200–400 mbar) were first determined. Subsequently, all possible combinations of individual methods were performed under the previously determined optimal conditions. The detoxification efficiency was evaluated by growth tests and quantification of selected model substances via gas chromatographic (GC) analysis.

### Single methods

#### Enzymatic laccase treatment

**Fig. 1:**
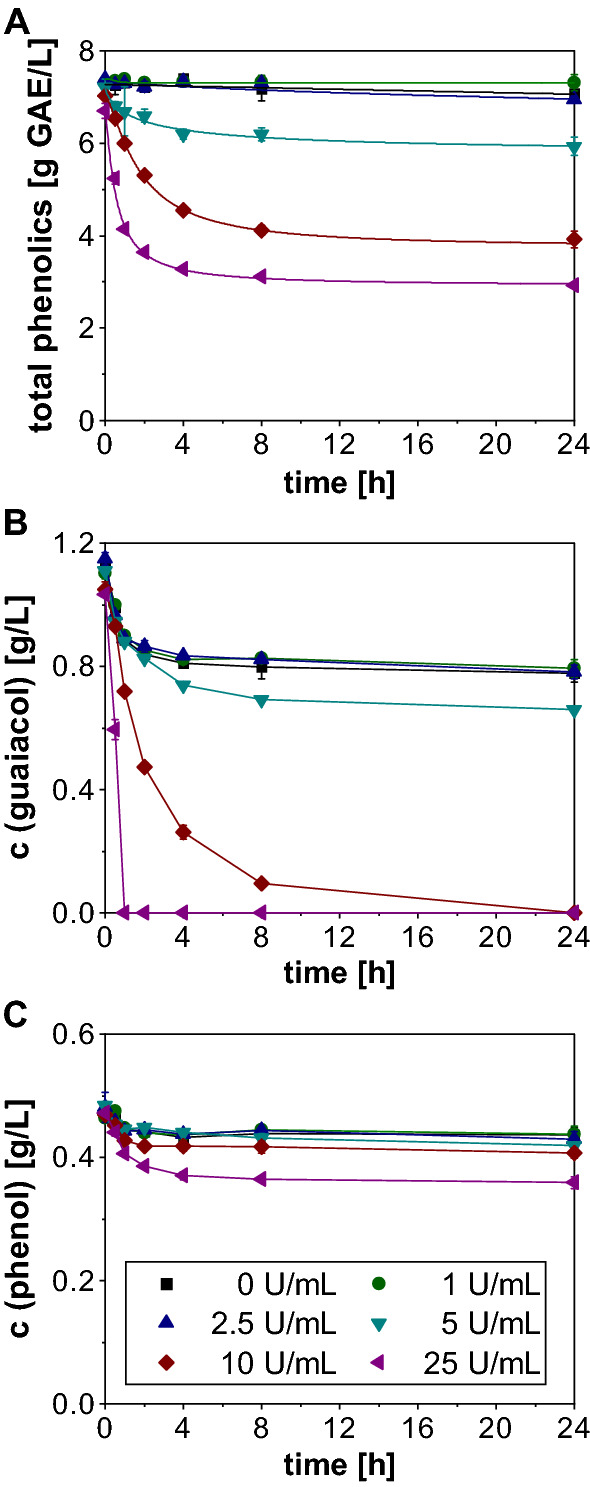
Removal of phenolic substances from PAC during 24 h treatment with different concentrations of *T. versicolor* laccase. The concentrations of total phenolics (**A**) were determined via Folin Ciocalteu (FC) analysis and expressed as g GAE/L. The single phenolic model compounds phenol (**B**) and guaiacol (**C**) were analyzed via GC. The experiments were performed in duplicates and the graph shows the mean values and the standard deviations as error bars

In a first experiment, the optimum enzyme concentration and duration for the laccase treatment were determined. Figure 1A shows that the initial total phenol concentration in the untreated PAC was 7.96 ± 0.04 g gallic acid equivalents (GAE)/L and only marginally decreased during the incubation period. The same behavior was observed for laccase concentrations of 1 and 2.5 U/mL. Only when 5 U/mL or more were applied a considerable decrease in the amount of total phenols became apparent. Even in the initial samples, a slight decrease in the phenol content was observed, indicating that the enzymatic reaction was quite fast. This assumption is supported by the almost linear decline during the first hour of incubation. Afterwards, the curves progressively flattened and the concentrations of the total phenolics remained largely constant during the last 8 h of treatment. The final titer ranged from 5.93 ± 0.20 g GAE/L for 5 U/mL to values of 3.92 ± 0.18 g GAE/L and 2.92 ± 0.05 g GAE/L when enzyme amounts of 10 and 25 U/mL were used. This corresponds to a percentage removal of 19.32%, 46.67% and 60.27% and shows that even with the highest laccase concentration the phenolics were not completely eliminated.

This observation was also confirmed by the GC analyses of the two phenolic model substances phenol and guaiacol (Fig. 1B and C). Whereas a complete removal of guaiacol was achieved after 24 h and 1 h when the PAC was treated with 10 U/mL and 25 U/mL laccase, respectively, the phenol concentration was only slightly affected by the enzyme addition. Solely for the highest laccase concentration, a considerable removal of 17.8% phenol was observed after 24 h. Thus, the results show that the removal of phenolics does not only strongly depend on the enzyme concentration, but also on the phenolic compound.

**Fig. 2:**
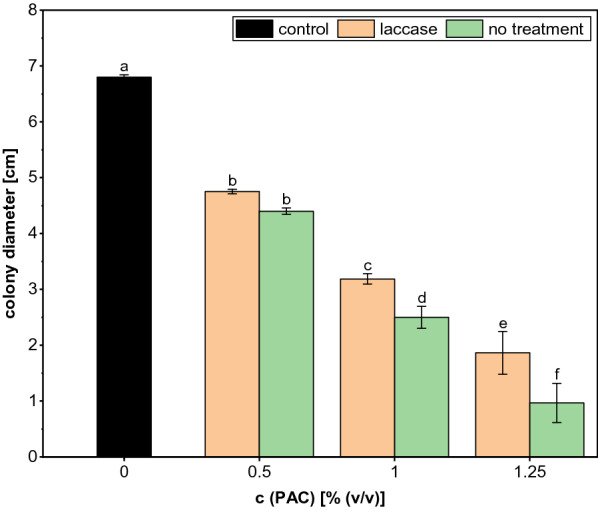
Colony diameters of *A. oryzae* after 5 days of growth on agar plates containing different contents of PAC treated with 25 U/mL *T. versicolor* laccase for 24 h. The experiment was performed in quadruplicates and the graph shows the mean values and the standard deviations as error bars

For the growth test, the highest enzyme concentration of 25 U/mL and the maximum incubation time of 24 h were chosen (Fig. 2). The fungal colony diameters were larger on plates that contained laccase treated PAC than on those with untreated condensate. However, growth was still strongly inhibited compared to the control without PAC and the overall tolerance level did not exceed 1.25%. Due to its ineffectiveness, laccase treatment was therefore not considered for the combinations of different treatment methods.

#### Activated carbon treatment

**Fig. 3:**
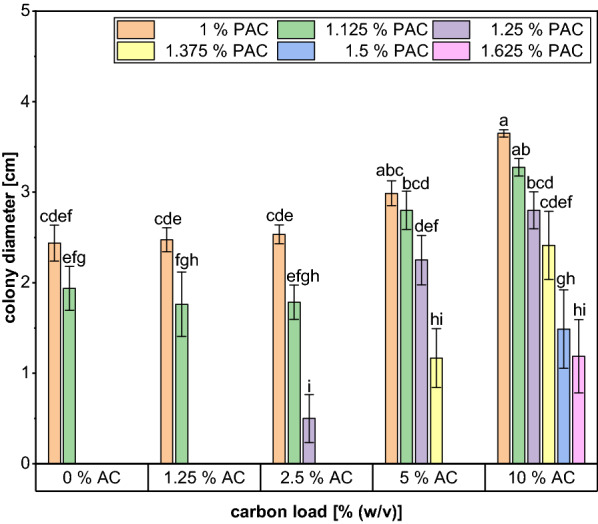
Colony diameters of *A. oryzae* after 5 days of growth on agar plates containing different volumetric contents of PAC treated with increasing carbon loads for 1 h. The experiment was performed in quadruplicates and the graph shows the mean values and the standard deviations as error bars

**Fig. 4:**
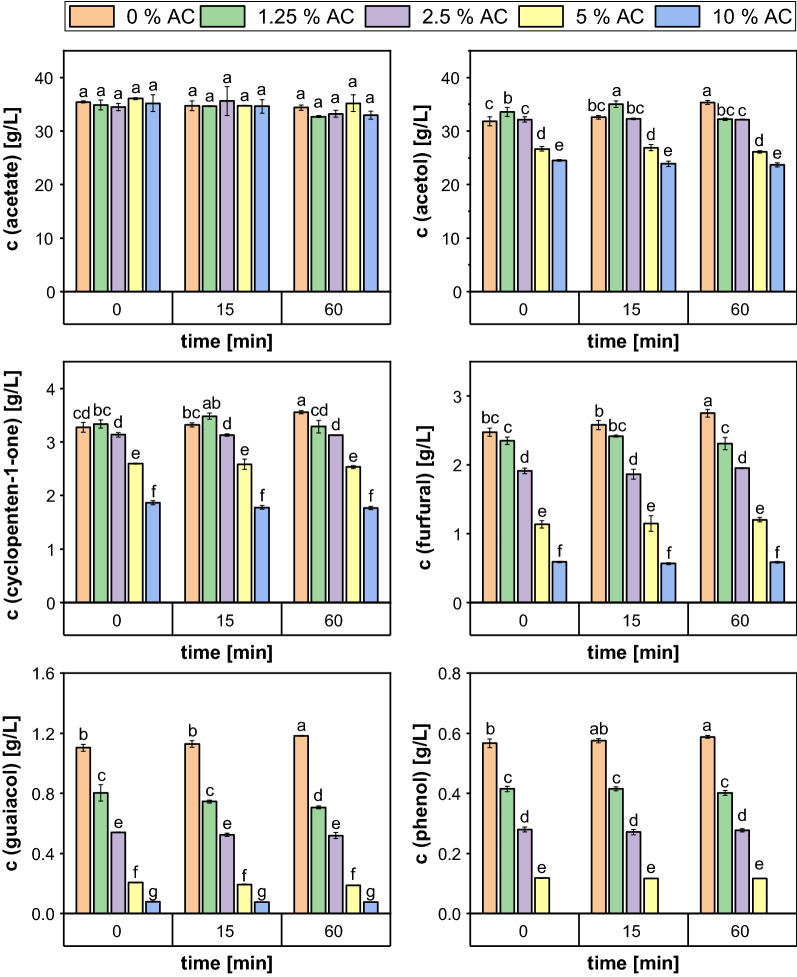
Temporal change in concentration of selected PAC compounds during treatment with increasing carbon loads. The data represent mean values of two replicates and error bars indicate the standard deviation

To determine the optimum concentration of activated carbon required for effective PAC detoxification carbon loads of 0–10% (w/v) were tested. As displayed in Fig. 3, only 1.125% PAC was tolerated when the condensate did not undergo any additional treatment besides a pH adjustment and subsequent filtration. Detoxification with 1.25% activated carbon did also not lead to any growth on higher PAC concentrations, whereas for carbon loads ≥ 2.5% an enhanced fungal PAC tolerance was detected. The growth limit of *A. oryzae* increased to 1.25%, 1.375% and 1.625% when the PAC was treated with 2.5%, 5% and 10% activated carbon, respectively. Furthermore, colony diameters increased for the same PAC content when applying higher activated carbon concentrations. Consequently, the best results were achieved using the highest amount of carbon leading to an improvement of 30.77% compared to the untreated control. The GC analysis revealed that the treatment resulted in at least a partial removal of all inhibitory compounds (Fig. 4). Interestingly, the lowest concentrations of the inhibitors were already reached immediately after the addition of the carbon and longer incubation times did not lead to any further improvement, which indicates that the adsorption to the char particles is a rather fast process. In addition, the effectiveness of the activated carbon treatment was highly dependent on the inhibitory PAC component. For acetol, 2-cyclopenten-1-one and furfural only slight changes in the concentration were detected after treatment with 1.25% AC, which coincides with the results of the growth test. By contrast, a notable removal of the phenolic compounds already occurred at the lowest carbon load applied, as their concentration was reduced by about 30%. Moreover, Fig. 4 shows that the concentration of the inhibitory compounds was further decreased by using higher carbon contents. In case of phenol, the use of 10% carbon even resulted in a complete removal of the compound and also the concentration of guaiacol was decreased by 93.4%. Although the concentration of the ketones and aldehydes was not reduced to such a high extent, a carbon load of 10% was still the most effective condition for the removal of these compounds as the concentration of acetol, 2-cyclopenten-1-one and furfural decreased by 29.5%; 48.3% and 77.7%, respectively. Only the amount of acetic acid remained relatively constant at an average concentration of 34.44 ± 1.40 g/L throughout the incubation period regardless of the carbon loads applied. However, this can be seen as a positive result as the acid is considered the most promising C-source for the fungal growth contained in the PAC.

Since the treatment with 10% carbon showed the best removal efficiency without affecting the acetate content, it was chosen for further experiments and the incubation time was shortened to 10 min.

#### Overliming treatment at different temperatures

**Fig. 5:**
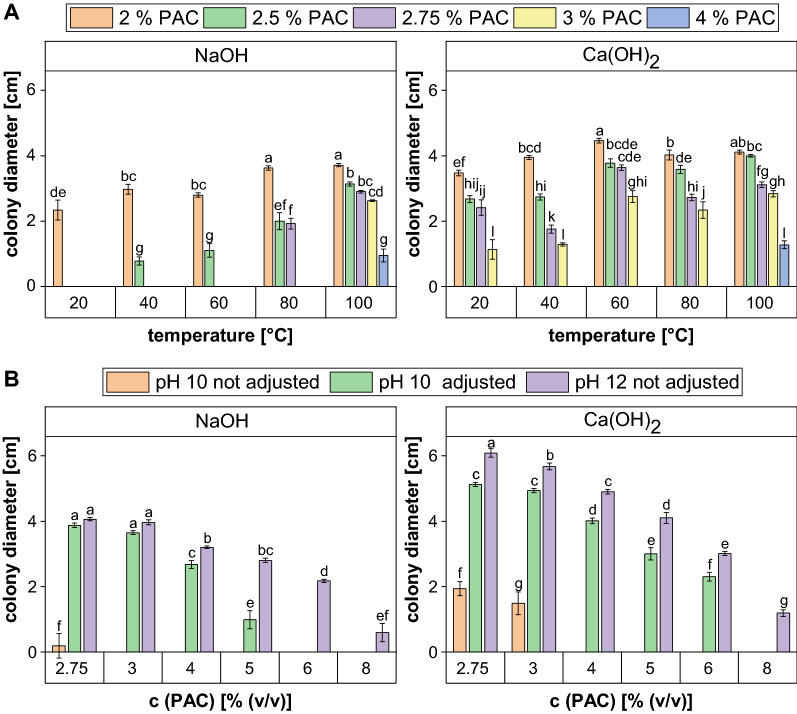
Colony diameters of *A. oryzae* after 5 days of growth on agar plates containing different contents of PAC treated with overliming at various temperatures for 4 h (**A**) and two different pH values for 90 min (**B**). In the pH experiment, two different initial pH values (pH 10 and 12) as well as manual adjustment to pH 10 throughout the process were tested for a fixed temperature of 80 °C. The experiment was performed in quadruplicates and the graph shows the mean values and the standard deviations as error bars

Growth tests revealed a general positive relation between the temperature applied for the overliming treatment and the fungal tolerance towards pretreated PAC (Fig. 5A). However, the temperature dependence appeared to be different for the two hydroxides. For NaOH, the tolerance level improved from 2% at room temperature to 2.5% and 2.75% when the incubation temperature was doubled to 40 °C and 80 °C, respectively. Thus, a clear correlation between the temperature and the detoxification efficiency was observed. Contrarily, the overliming treatment with Ca(OH)_2_ seemed to be already quite effective at low temperatures, as even with a treatment performed at 20 °C the fungus could tolerate PAC concentrations of 3%. This corresponds to 50% better growth compared to the NaOH overliming conducted under the same conditions and was still 8.33% better than the NaOH treatment at 80 °C.

However, this growth limit did not increase for higher temperatures up to 80 °C. Only when a temperature of 100 °C was used for the Ca(OH)_2_ overliming treatment the fungal tolerance was further improved to 4%. The same tolerance level was also achieved for NaOH at that temperature, thus leading to the assumption that the influence of the metal ion that became apparent at low temperatures was compensated at 100 °C. This assumption is also confirmed by GC analysis of the PAC compounds after 4 h of treatment at 100 °C, as it revealed comparable concentrations for both hydroxides at these conditions (Table 1). Only two inhibitory compounds, guaiacol and 2-cyclopenten-1-one, differed notably in their final concentrations. For guaiacol, a slightly higher concentration of 0.67 ± 0.01 g/L was observed in the NaOH-treated PAC, but for both hydroxides its content dropped well below the growth limit of 1% that was determined by Dörsam et al. [19]. Regarding cyclopenten-1-one, the concentration of 1.22 ± 0.01 g/L was 9% higher in the Ca(OH)_2_ treated PAC than when sodium was used. Although the treatment already resulted in a reduction of the 2-cyclopenten-1-one content to about one third of the concentration in the original PAC, the fungal growth limit of 0.0625 g/L was still significantly exceeded in both cases, which was also reflected in the results of the growth test. While the concentration of all inhibitory substances decreased during the overliming treatment, acetic acid concentrations seemingly increased (Table 1), partly due to evaporation effects.

**Table 1:** GC analysis of pyrolytic aqueous condensate (PAC) treated with overliming at different conditions

Substance	*A. oryzae* growth limit [g/L]^a^	c in original PAC [g/L]^b^	c [g/L] in PAC after treatment at 100 °C and the following conditions…			
			Duration [h]	4	4	1.5
			Initial pH	10	10	12
			Hydroxide	Ca(OH)2	NaOH	NaOH
Acetate	> 70	29.76		40.51 ± 0.19	38.35 ± 0.33	41.24 ± 1.87
Acetol	15	51.46		9.99 ± 0.91	11.50 ± 0.75	6.31 ± 0.42
Guaiacol	1	1.80		0.55 ± 0.01	0.67 ± 0.01	0.48 ± 0.00
Furfural	0.3	2.94		0.22 ± 0.03	0.31 ± 0.06	0.12 ± 0.00
Phenol	0.7	0.64		0.44 ± 0.00	0.46 ± 0.01	0.4 ± 0.00
2-Cyclopenten-1-one	0.0625	3.40		1.22 ± 0.01	1.11 ± 0.03	0.45 ± 0.00

^a^Determined by Dörsam et al. [19] as % (w/v). The concentration was converted into g/L assuming the density of water [2]. The only exception was acetate, for which, according to the results of Kövilein et al. [42], no growth limit was determined so far

^b^GC/MS analysis performed by Thünen Institute of Wood Research in Hamburg for PAC that was neither pH adjusted nor filtered

**Table 2:** GC analysis of pyrolytic aqueous condensate (PAC) treated with different combinations of detoxification methods

Combination^a^	c (PAC compound) [g/L]					
	Acetate	Acetol	2-Cyclopenten-1-one	Furfural	Guaiacol	Phenol
Untreated	35.17 ± 0.13	33.58 ± 1.86	3.42 ± 0.13	2.63 ± 0.15	1.15 ± 0.04	0.58 ± 0.01
Growth limit^b^	> 70	15	0.0625	0.3	1	0.7
RE	36.06	6.89	0.09	0.21	0.16	0.06
RE + OL	39.97	3.95	0.08	0.00	0.16	0.06
RE + OL + AC	39.02	2.96	0.00	0.00	0.14	0.00
RE	35.17	6.34	0.09	0.20	0.15	0.06
RE + AC	35.40	5.06	0.00	0.00	0.14	0.00
RE + AC + OL	40.07	3.54	0.00	0.00	0.00	0.00
OL	42.56	6.61	0.44	0.12	0.48	0.41
OL + RE	42.83	2.76	0.11	0.23	0.22	0.09
OL + RE + AC	43.49	2.10	0.07	0.15	0.14	0.11
OL	39.92	6.01	0.45	0.12	0.48	0.41
OL + AC	42.40	5.79	0.30	0.04	0.21	0.06
OL + AC + RE	42.41	1.96	0.08	0.00	0.15	0.00
AC	35.81	21.64	1.62	0.40	0.16	0.00
AC + RE	34.59	5.69	0.24	0.10	0.14	0.00
AC + RE + OL	44.43	3.58	0.16	0.00	0.13	0.00
AC	35.43	21.93	1.64	0.40	0.14	0.00
AC + OL	41.84	14.15	0.61	0.05	0.14	0.00
AC + OL + RE	41.22	6.18	0.12	0.00	0.00	0.00

^a^All possible combinations of rotary evaporation (RE); overliming (OL), and activated carbon treatments (AC) were performed

^b^Determined by Dörsam et al. [19] as % (w/v). The concentration was converted into g/L assuming the density of water [2]. The only exception was acetate, for which, according to the results of Kövilein et al. [42], no growth limit was determined so far

In general, the results show that a temperature of 100 °C serves best for PAC detoxification. Hence, this temperature was chosen for the overliming treatments performed in the combination experiments. However, a PAC tolerance of 4% is still far too low to enable its utilization as sole substrate for fungal growth and a further development of the treatment procedure is required.

#### Overliming treatment using different pH strategies

As it can be seen from the pH curve during the temperature experiment (Additional file 2: Fig. S1) the pH drops back into the neutral range very quickly, especially when high temperatures are applied. Consequently, it was assumed that the hydroxide ions were depleted and that a higher supply could further enhance detoxification. Therefore, two different ways to increase the hydroxide concentration were evaluated: raising the initial pH to 12 or adjusting the pH to a value of 10 manually throughout the incubation period. Figure 5B shows that both strategies led to a remarkable increase in the PAC tolerance of *A. oryzae* as for NaOH the maximum tolerated concentration could be enhanced to 5% for the pH adjusted PAC and even to 8% with an initial pH of 12. This corresponds to a 45% and 66% improvement compared to the standard procedure performed at an initial pH of 10.

Again, slightly better results were obtained for Ca(OH)_2_ as for the pH adjusted PAC the maximum concentration tolerated by the fungus was 6%. However, for the pH strategy that served the best (initial pH of 12) the effect of the hydroxide seemed to be neglectable again as both hydroxide treatments led to the same PAC tolerance of 8%. Therefore, this pH strategy was chosen for the subsequent combination experiments and NaOH was selected to be the favored hydroxide in order to reduce the risk of precipitation of medium components. Due to the upper temperature limit of the pH probe used, the pH experiment had to be performed at 80 °C, even though a temperature of 100 °C was previously identified to be ideal. A combination of the optimum conditions for temperature and pH resulted in an even higher tolerated PAC concentration of 12.5% (data not shown) and the GC results are shown in Table 1. The results underline that the pH is of particular importance for an efficient overliming treatment, since the concentration of all inhibitory compound measured was further reduced by the pH optimization in an even shorter reaction time. In particular, the amount of acetol, furfural and 2-cyclopenten-1-one was considerably reduced by 45.1%, 61.3% and 59.5%, respectively, compared to the overliming treatment with an initial pH of 10. In contrast, only minor decreases in guaiacol and phenol were observed, leading to the conclusion that overliming is not the preferred method for the removal of phenolic substances.

#### Rotary evaporation for removal of inhibitory compounds

**Fig. 6:**
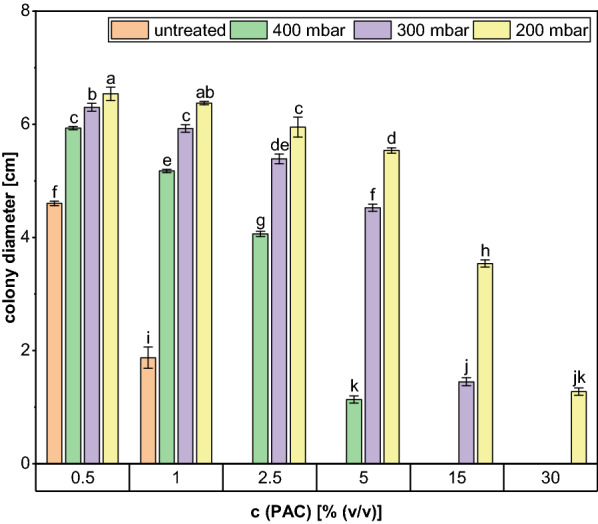
Colony diameters of *A. oryzae* after 5 days of growth on agar plates containing different volumetric contents of PAC treated with 4 h rotary evaporation at different pressures and 80 °C. The data represent mean values of quadruplicates and the error bars indicate the standard deviation

In this experiment different pressures were selected for a 4 h rotary evaporation at 80 °C and the maximum concentration that was still tolerated by *A. oryzae* was enhanced to 5%, 15% and even 30% when the condensate was treated at 400 mbar, 300 mbar and 200 mbar, respectively (Fig. 6). These results indicate a clear dependence of the detoxification efficiency on the pressure applied for the evaporation.

**Fig. 7:**
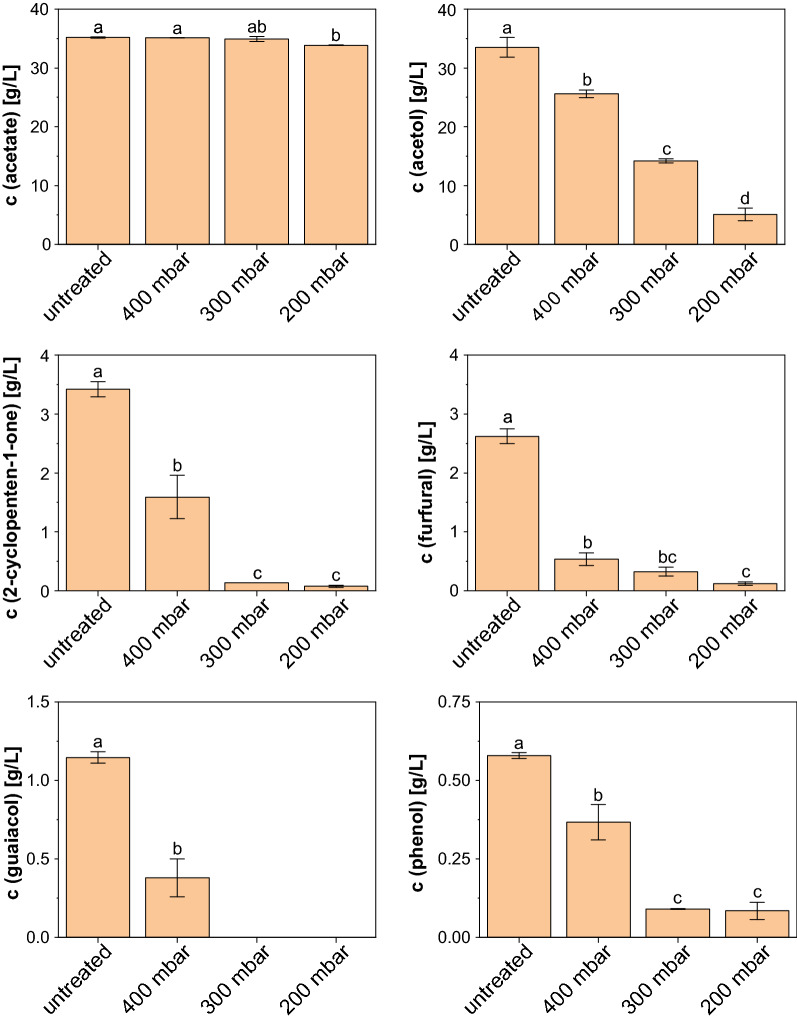
Concentration of selected PAC compounds after 4 h treatment with rotary evaporation at different pressures and 80 °C. The data represent mean values of two replicates and error bars indicate the standard deviation

The growth results are supported by the GC analysis since the concentration of every measured inhibitory PAC compound decreased remarkably. Even at 400 mbar the removal of the toxic compounds ranged from 23.6% for acetol to a maximum of 79.4% for furfural compared to the control (Fig. 7). The detoxification results were further improved when lower pressures were maintained throughout the evaporation procedure. A reduction of the pressure to 300 mbar resulted in the complete removal of the phenolic compound guaiacol and also the concentration of 2-cyclopenten-1-one was decreased remarkably by 95.9% to 0.14 g/L. However, the content of the latter and the remaining 0.32 ± 0.08 g/L of furfural still exceeded the growth limit of *A. oryzae* (Table 1). By further decreasing the pressure to 200 mbar, it was possible to obtain an additional removal of almost every substance, except for phenol. The concentrations of furfural and 2-cyclopenten-1-one were now determined to be 0.12 ± 0.03 g/L and 0.08 ± 0.02 g/L, and thus were below or at least close to the growth limit of the fungus. As already observed in the previous experiments, the content of acetic acid remained largely unchanged at an average of 35.07 ± 0.45 g/L. Only when a pressure of 200 mbar was applied, a significant decrease (*p* = 0.015) to a concentration of 33.86 ± 0.04 g/L was observed. However, due to the fact that the best detoxification results were obtained for this condition, it was chosen for the following combination experiment.

#### Combinations of methods

**Fig. 8:**
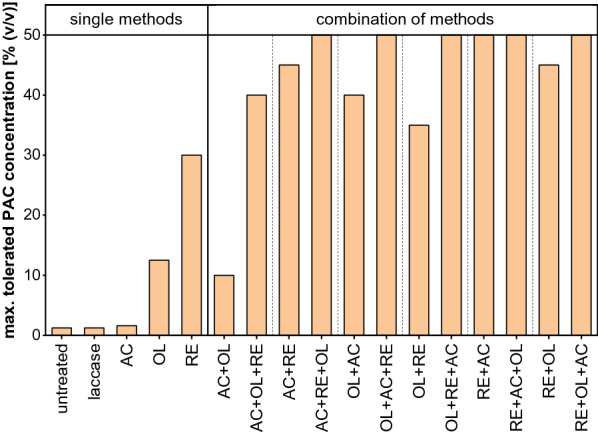
Maximum tolerance of *A. oryzae* on agar plates containing PAC obtained after single treatment with laccase, activated carbon (AC), overliming (OL) or rotary evaporation (RE) as well as combinations of the latter three detoxification methods

The previous sections showed that the application of single methods is not sufficient to enable fungal growth on the pure condensate. Therefore, it was evaluated whether combinations of activated carbon (AC), overliming (OL) and rotary evaporation (RE) treatments further improve the fungal PAC tolerance (Fig. 8). Every possible combination of two and three methods was performed and all except the combination of AC + OL resulted in a higher tolerance than achieved for the single methods. The data also show that the improved growth of *A. oryzae* that was obtained after the combination of two methods was further enhanced when a subsequent third treatment was conducted. Except for AC + OL + RE, all combinations of three methods allowed for a fungal growth on 50% PAC, which was the maximum concentration that could be tested on agar plates. Consequently, these data were not suitable to evaluate the role of the order in which the individual detoxification procedures should be performed.

Nevertheless, the results obtained for the combinations of two methods indicate that the order of the treatment methods seems to be vitally important. For instance, the combination AC + OL led to a tolerance of only 10%, whereas performing the combination in the reverse order resulted in a fungal growth on up to 40% PAC. A similar observation was made for the combinations of AC + RE and RE + AC, which leads to the assumption that the activated carbon treatment should be conducted at the end of the detoxification procedure. Conversely, the latter example also indicates that is has a positive effect on the PAC detoxification to perform the rotary evaporation in the beginning. This hypothesis is further supported by the comparison of the combinations OL + RE and RE + OL, since the tolerated PAC concentration was 10% higher for the latter combination. Performing RE in the beginning and AC in the end (RE + AC) gave the best results among all combinations of two single methods as a growth on the maximum concentration of 50% PAC was observed.

This raised the question of whether an additional OL treatment is even needed to enable the utilization of the pure condensate as substrate for fungal growth. Therefore, RE + AC and the two best performing combinations of three methods according to their colony diameter and morphological appearance were selected for a shake-flask cultivation using 100% PAC (data not shown). The results revealed that *A. oryzae* was capable of growth only in the PAC treated with RE + OL + AC, so this combination was henceforth considered as optimal detoxification procedure.

To allow for a better understanding of the growth data, samples were taken after each individual treatment step of the respective combination and analyzed by GC (Table 2). The data obtained after the first step of each combination are largely in agreement with the findings of the single method experiments performed in the previous sections. It was again confirmed that, in contrast to overliming, activated carbon treatment is suitable for removing phenolic compounds. In addition, the concentration of acetic acid remained largely unchanged after AC and RE treatments, while it even increased for OL. This demonstrates that the treatment results are quite reproducible despite the complex and variable composition of the PAC.

Regarding the combination of two methods, the lowest fungal tolerance was observed for AC + OL. The GC results show that after the initial AC treatment, phenol was completely removed. However, 0.40 g/L furfural and 1.62 g/L 2-cyclopenten-1-one were still present in the PAC. Although the subsequent OL procedure led to a further removal of the analyzed inhibitors, the concentrations of 2-cyclopenten-1-one and acetol were still even higher than when OL was performed as sole treatment. This leads to the assumption that OL is impaired by the preceding carbon adsorption and confirms that the AC treatment should better be conducted at the end of the detoxification procedure. In contrast, performing RE in the beginning resulted in very low concentrations of every inhibitory compound and for the best performing combination of two methods (RE + AC), even a complete removal of furfural and 2-cyclopenten-1-one was observed.

The same observations were made for both of the combinations of three methods that started with a RE treatment. One of these detoxification procedures, namely RE + OL + AC, was the only one that enabled *A. oryzae* to grow on 100% PAC. However, the GC analysis of only six PAC compounds cannot fully explain why RE + AC and RE + AC + OL combinations were less effective, since even for these combinations the concentrations of all the inhibitors measured was below the growth limit of *A. oryzae.*

#### *Aspergillus oryzae* cultivation

**Fig. 9:**
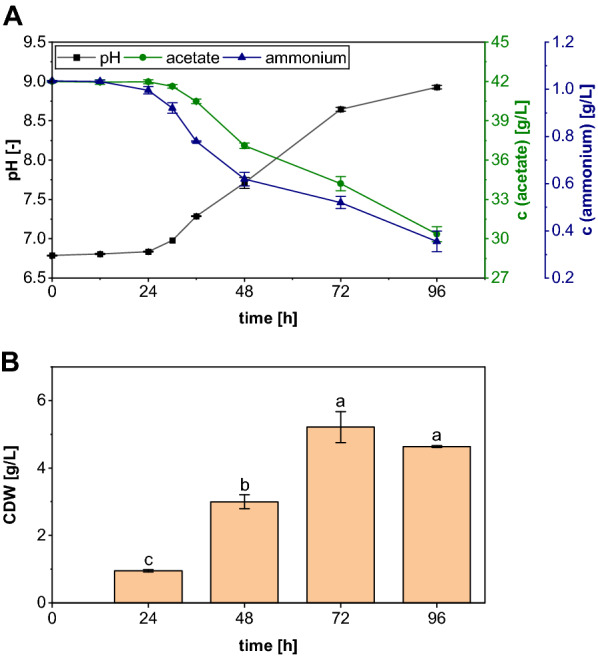
Shake-flask cultivations of *A. oryzae* in medium containing 100% PAC detoxified by a combination of rotary evaporation, overliming and activated carbon. The temporal changes in the substrate concentrations and pH (**A**) as well as the CDW (**B**) were tracked for 96 h. The data represent mean values of triplicates and the error bars show the standard deviation

The PAC obtained after treatment with the best performing detoxification procedure was now utilized for the evaluation of the growth and substrate consumption of *A. oryzae* cultivated in shake flasks containing 100% treated PAC (Fig. 9). It can be seen that the initial acetate concentration was 42.00 ± 0.07 g/L and remained largely unchanged during the first 24 h of cultivation. The same observation was made for the pH and the ammonium concentration. However, Fig. 9B shows that a cell dry weight (CDW) of 0.95 ± 0.03 g/L was already obtained at this stage of fermentation, indicating that either additional usable substrates are present in the PAC or that the fungus consumed the nutrient reserves that were still contained in the spores. After the first adaptation phase, the pH increased considerably due to the incipient acetate consumption. The concentration of the carbon source decreased by 4.91 g/L and also the amount of ammonium was reduced to 0.62 ± 0.03 g/L during the exponential growth phase (24–48 h). The high substrate consumption led to a sharp increase in biomass formation, as a tripling of the CDW to 3.00 ± 0.21 g/L was detected. Although this was followed by an incipient flattening of the substrate and pH curves, the biomass continued to increase and the maximum CDW of 5.21 ± 0.46 g/L was reached after 72 h. Only in the last 24 h of cultivation a slight, although not significant (*p* = 0.07) decrease in the biomass concentration to 4.63 ± 0.03 g/L was observed. Thus, the fungus gradually entered the stationary growth phase, even though 30.35 ± 0.57 g/L acetate and 0.36 ± 0.04 g/L ammonium were still present at the end of the cultivation, indicating that another medium component became limiting. Nevertheless, the results of the shake-flask experiment show that with the choice of an appropriate detoxification procedure, it is possible to use the PAC as the sole substrate for the growth of *A. oryzae*.

## Discussion

### Single methods

#### Laccase

In contrast to the other tested methods, laccase treatment did not improve fungal PAC tolerance even though at least a partial removal of phenolics was detected via FC assay and GC. The mechanism of removal is based on the oxidation of phenolic compounds, which is catalyzed by the copper-containing enzyme and is accompanied by the simultaneous reduction of oxygen to water. This oxidation is a one-electron reaction that leads to the formation of a free phenoxy radical, which can undergo further non-enzymatic reactions, including polymerization with other phenolic molecules [43]. Since these polymers are insoluble in aqueous solutions, they form reddish to dark brown precipitates as it was also observed in the current study. This observation confirms that the enzyme was active under the selected conditions as previously reported by Kurniawati et al. [44]. However, since they used defined mixtures with only 500 µM pure phenol for their experiments, it is rather difficult to compare their results with the complex PAC, containing a large variety and high concentrations of phenolic substances.

Much more comparable conditions underlay the work of Jönsson et al., as they used ligninolytic enzymes from *T. versicolor* for detoxification of wood hydrolysates and were also not able to detect a complete removal of all phenolic substances [34]. Furthermore, they found the concentration of guaiacol to be decreased by 97%, whereas 34% of phenol was still left in the hydrolysate. This observation coincides with the results obtained in our work and is further supported by Kolb et al., who classified phenolic compounds into three different laccase reaction groups and identified guaiacol to be a molecule that is eliminated relatively fast [35]. The occurrence of different reaction groups can be explained by the fact that the effectiveness of laccase appears to be dependent on the chemical structure of the phenolic substrates. For instance, Jarosz-Wilkołazka et al. revealed that the enzymatic conversion of guaiacol by laccase originating from *Cerrena unicolor* appeared to be faster than for phenol due to its methoxy substituent in ortho-position [45].

Additionally, enzymatic removal of phenolic compounds might be inhibited by non-phenolic PAC components, as it was already shown in literature that laccase activity can be impaired by carboxylic acids like acetic and propionic acid [46, 47]. However, Kreuter et al. stated that inhibition was only observed for the acid and not for its salt form. Since the enzymatic treatment of the PAC was performed at pH 6.5 it can be assumed that most of the carboxylic acids were in a dissociated state making an inhibition rather unlikely. Not only the enzyme, but also the FC assay can be negatively affected by acetate and other interfering substances [48, 49], which might explain why some differences in the FC and the GC analysis results occurred. While according to the FC assay the total phenolics content was only marginally decreased in the negative control, a considerable decline was observed for the GC measurements, especially for guaiacol. Such discrepancy between the FC measurement and the analysis of individual phenols was already noted earlier by Kolb et al., however, no explanation was given [35].

According to the FC analysis, we were not able to remove the total phenolics completely even with 25 U/mL of enzyme. Presumably, higher laccase concentrations could thus further improve the elimination of phenolic substances, but an enzymatic treatment of the PAC is relatively expensive. Furthermore, considering the growth limits of *A. oryzae* for single PAC components according to Dörsam et al. [19], it can be assumed that even a complete removal of phenolics would most likely not lead to a substantial improvement of the fungal PAC tolerance, as even the content of phenolics in untreated PAC is already close to the growth limit. In their work, the strongest inhibition of the fungal growth was caused by cyclic aldehydes and ketones like furfural and especially 2-cyclopenten-1-one. Therefore, a biological detoxification that aims at a valorization of the PAC using *A. oryzae* should primarily target these molecules. For instance, there have been several approaches for the biological removal of furfural and other furanic compounds from lignocellulose degradation products using fungi [32, 50–52], but also bacterial strains [53–55].

#### Activated carbon

The application of activated carbon especially for the effective removal of phenolic substances from contaminated wastewater has been described extensively in literature [56–59] and could thus represent a more cost-effective alternative to laccase.

Indeed, the treatment of the PAC with 10% (w/v) activated carbon reduced the concentration of guaiacol by 96% and even a complete removal of phenol was observed. This indicates that the adsorption of phenolics on the carbon surface depends at least partly on their chemical structure. Mattson et al. suggested that phenolic substances adsorb on activated carbon via the formation of a ‘‘donor–acceptor complex’’ between carbonyl oxygen groups on the carbon surface acting as electron donor, and the aromatic ring of the adsorbate acting as acceptor [60]. In contrast to phenol, guaiacol contains an additional methoxy group increasing the electron density in the aromatic ring. This might lead to a weaker binding of the guaiacol and give a potential explanation for the incomplete removal of the methoxyphenol. Moreover, the PAC does not only contain the two phenolic species measured, but rather a complex mixture of phenolic components that can impair each other in their adsorption behavior [61].

In contrast to detoxification with laccase, treatment with activated carbon is less specific and also removes other non-phenolic PAC components that were identified to be more harmful to the fungus [19]. For example, furfural content was decreased by 78% after 1 h of treatment with 10% carbon. Several other studies have evaluated the potential of activated carbon for removal of furfural as a well-known microbial inhibitor [62–65]. However, these studies were based on diluted furfural solutions, which makes it difficult to compare the results with those obtained for the PAC, since its components can influence each other in their adsorption behavior. For instance, it is known that a competitive adsorption of furfural and phenolic compounds exists with the latter having a higher potential to bind to the carbon surface and even to displace the weakly bound furfural [66].

Therefore, the adsorption of inhibitory compounds on activated carbon has to be evaluated in authentic liquid lignocellulose degradation products. Lee et al. performed a treatment of wood hydrolysate with carbon loads of 1–10% (w/v), which is a comparable range to the one tested in our study. They analyzed the impact of the carbon content on the elimination of sugars, carboxylic acids and the furans HMF and furfural observing an improved removal with increasing carbon loads [23]. This coincides with the results obtained in our work and can most likely be attributed to the increase in the carbon surface area and the higher number of adsorption sites. In contrast to our study, an almost complete removal of both furans was detected for concentrations ≥ 2.5% activated carbon that might potentially be explained by the applied elevated temperature of 50 °C. However, previous findings on the effect of the temperature on furfural removal were partly contradictory, as in some studies an improvement in detoxification with increasing temperature was reported due to a higher molecular motion [67], whereas others claimed that the adsorption of furfural is an exothermic process that is impaired by increasing temperatures [62, 65]. Another explanation for the discrepancy could be that hydrolysates often contain less inhibitory compounds than pyrolytic condensates since the reaction conditions are milder, so fewer compounds can interfere with the adsorption of furans.

Liang et al. conducted their detoxification experiments with an acid-rich fraction of the fast pyrolysis of mixed softwood and provided a comprehensive insight into the adsorption of various inhibitors [22]. In particular, the results obtained for the adsorption behavior of acetol are of great importance for PAC valorization with *Aspergillus oryzae*, since it constitutes the main component of the condensate, but has an inhibitory effect already at concentrations of ≥ 1.5% [19]. In addition, acetol can be seen as a model compound for the adsorption of other ketones contained in the PAC. Liang et al. did not observe a complete removal of acetol, even though they used high activated carbon loads of up to 0.7 g/mL [≙ 70% (w/v)] and also in our work the adsorption of ketones like acetol and 2-cyclopenten-1-one was the weakest with only 29.5% and 48.3% being removed.

In their study, Liang et al. also evaluated the impact of the pH on the adsorption of the model compounds and found that the binding of carboxylic acids in particular was strongly dependent on the pH of the solution. While they observed significant removal of acetic acid and formic acid at low pH values, almost no decrease in concentration was detected at pH > 6. This is in accordance with the results obtained here since the experiments were performed at pH 6.5 and the acetic acid concentration remained largely constant at 34.44 ± 1.40 g/L. The pH dependence can most likely be explained by the functional groups that are attached to the surface of the carbon. These groups can be acidic, neutral or alkaline and give the carbon surface an amphoteric character affecting its properties depending on the pH of the surrounding solution. When the pH of the PAC is higher than the pK_a_ of acetic acid (pK_a_ = 4.75), the acid becomes dissociated and its adsorption is dependent on the charge of the carbon surface [68]. However, a rise in pH also results in an increasing number of negatively charged functional groups on the carbon surface and causes an electrostatic repulsion between the carboxylate and the surface groups [68, 69].

Despite the partial removal of all analyzed inhibitors, the fungal tolerance to the PAC was only slightly increased from 1.125 to 1.625% after 1 h of treatment with 10% carbon. As it was shown in this work, prolonging the contact time between carbon and PAC did not result in an improved inhibitor removal. In contrast, chemical treatment of the activated carbon could be a potential strategy to improve detoxification, as it increases the number of functional groups on the carbon surface and its microporosity [70]. Additionally, the utilization of even higher carbon loads seems to be a possible way to improve detoxification, as our results show a clear correlation between the amount of activated carbon used and the removal of toxins. However, an increase in the carbon content also results in a higher viscosity of the suspension, making it increasingly difficult to ensure sufficient mixing. Moreover, it results in greater losses of PAC and a larger amount of saturated carbon that needs to be disposed after the treatment. For this reason, various studies have already been published on the regeneration and repeated use of activated carbon in order to reduce the amount of waste produced (for example reviewed in [71]). Reuse of carbon can only be considered as an alternative for discarding if the regeneration costs do not greatly exceed the disposal costs and if no additional waste streams such as solvents are generated during the process. Alternatively, the char fraction that is already produced during the pyrolysis process could be used for activated carbon production. For the bioliq^®^ process, the feasibility of this alternative char utilization was already assessed, but primarily with the objective of selling the activated carbon to cover the process costs [7] whereas in the work of del Campo et al. the carbon obtained was used directly for the detoxification of the water-soluble fraction of the pyrolysis oil aiming at its fermentative valorization [72].

#### Overliming

The detoxification via overliming is based on the chemical reaction of the inhibitors to form less harmful or even non-toxic substances. Even though extensive literature on overliming exists, especially for the detoxification of hydrolysates [73–75], most of the studies contain little information about the chemical reactions underlying this detoxification procedure. Instead, they focus on the decrease in the concentration of individual selected inhibitors as a function of various parameters such as pH, temperature, reaction time, and the type of hydroxide to find the optimum conditions for detoxification.

In our study, a partial removal of all measured toxins was observed, although it appeared that overliming was not equally effective for all PAC components. For example, the treatment at pH 12 and 100 °C reduced the concentration of phenol by only ~ 38%. Similarly, Millati et al. tested the effects of different pH values, temperatures and treatment durations on the overliming of a dilute-acid hydrolysate and found the phenol concentration to be only marginally effected by the conditions applied [76]. By contrast, they detected a strong decrease in furfural and HMF concentration and also a positive correlation between pH and the removal of furans. In our work, the concentration of furfural was also considerably reduced by 90% compared to the original PAC when an initial pH of 10 was applied and was even further reduced to 4% of the initial concentration when a starting pH of 12 was chosen. The drastic reduction in furfural concentration is not particularly surprising, since the presence of an aldehyde group and the heterocyclic structure of the furfural molecule enable a variety of chemical reactions [77]. Some of these reactions have already been shown to occur under strongly alkaline conditions, among them the resinification, in which furfural molecules polymerize to form a black insoluble residue [78]. The formation of dark precipitates was also observed during the overliming experiments performed in our study. However, resinification may not only occur between furfural molecules, but has also been observed between the aldehyde and phenol [79–81], which could provide a possible explanation for the 38% decrease in phenol concentration. In addition to reacting with phenol, Hronec et al. found that furfural may undergo aldol condensation reactions with cyclic ketones such as cyclopentanone, in which the components polymerize to form C15–C17 molecules and also oily insoluble intermediates were observed [82]. Cyclopentanone is a compound that was present in the PAC (Additional file 1: Table S1), but whose concentration was not tracked during the detoxification experiments, as it was found to be non-toxic to the fungus (data not shown). However, the reaction would also be conceivable for the highly inhibitory unsaturated ketone 2-cyclopenten-1-one, for which a remarkable removal of up to 87% was observed.

Some of the chemical reactions induced by the presence of hydroxide species can cause the formation of acids, which could potentially explain the pH drop observed during overliming (Additional file 2: Fig. S1). One of these reactions could be the intermolecular Cannizzaro reaction, in which two furfural molecules react to form furfuryl alcohol and furoic acid [83]. A reaction of acetol to lactic acid and pyruvic acid is also possible under alkaline conditions [84] and could therefore explain the reduction in the acetol concentration by up to 88%. However, the decline in pH may also be due to the observed increase in acetic acid, which was the only PAC component measured whose concentration was not reduced by the overliming. Although the unsuitability of this detoxification procedure to remove acetic acid has already been described several times [29, 30, 76], to our knowledge only one study mentions an increase, but attributes it to the degradation of sugar molecules [85]. Since the pyrolysis condensate used in the current work does not contain any sugars, this explanation does not apply here. Alternatively, the treatment temperature of 100 °C might have resulted in an increased evaporation and a partial escape of the hot vapors. But since the increase in concentration at this temperature was even higher when the initial pH was raised from 10 to 12, a chemical reaction can be inferred as the main cause of the observation. For instance, Machell et al. studied the effect of aqueous calcium and sodium hydroxide solutions on diacetyl and detected a formation of acetate without any apparent dependence on the type of hydroxide [86].

The GC analysis in our study also showed hardly any differences between the use of NaOH and Ca(OH)_2_ for the overliming treatment at 100 °C, which coincides with the growth results for *A. oryzae* at this temperature. Only for lower temperatures an enhanced growth was detected when overliming was performed with Ca(OH)_2_. An improved detoxification with Ca(OH)_2_ has already been described several times in literature [87–89] with Zhao et al. proposing that calcium ions can react with certain inhibitory compounds to form insoluble precipitants such as the calcium half-salt, PhO-Ca-OH [29].

Although the concentration of inhibitors was noticeably decreased by overliming treatment, the fungal growth limit towards PAC did not exceed 8%. This could possibly be explained by the fact that the 2-cyclopenten-1-one content was still significantly above the maximum concentration of 0.0625 g/L tolerated by the fungus (Table 1). However, the PAC also contains a large number of other substances that were not analyzed and whose toxic effects on the fungus are still unknown. Due to the variety of PAC components, the high energy input by heating and the high reactivity of the hydroxides, it can be assumed that many more reactions take place than those mentioned here. It is therefore conceivable that these reactions might even produce additional toxic compounds that prevent further increases in fungal tolerance.

#### Rotary evaporation

Among all the single methods tested, the rotary evaporation at 200 mbar was the most effective one since the concentration of all inhibitors was reduced to a level that is beneath or at least close to the growth limit of *A. oryzae*. For example, the concentration of the most toxic components 2-cyclopenten-1-one and furfural could be reduced by up to 97.7% and 95.4%, respectively, when the treatment was carried out at 200 mbar. That rotary evaporation is a suitable procedure to eliminate the latter from lignocellulosic degradation products has already been shown by Wilson et al. when they rotoevaporated an aspen wood hydrolysate at 55 °C to near dryness and detected a complete removal of furfural [90]. The same observation was made by Larsson et al. after they reduced the volume of their spruce hydrolysate by 90% via rotary evaporation [88]. In addition to the furfural removal, both groups observed at least a partial decrease in the acetic acid content after their treatments. This is not consistent with the findings of our evaporation experiments, where the concentration of acid remained largely constant. The main reason for the different results is probably the pH value of the hydrolysates. Since none of the papers described an adjustment of the pH before evaporation, it can be assumed that it was performed in the acidic range. Rodriges et al. described the pH dependence of the evaporation of acetic acid and found that the acid can only be removed in its undissociated, but not in its dissociated form [91]. Since the experiments in the current study were conducted at pH 6.5 most of the acid is dissociated to acetate. Another difference with our results was that both studies did not report a reduction of phenolics after evaporation of hydrolysates, whereas we noted a considerable decrease in the concentration of phenol by 86.2% and even a complete removal of guaiacol after 4 h evaporation at 200 mbar. Possibly, the temperature of 55 °C at which the experiments of Wilson et al. were carried out was not sufficient to enable a removal of phenolics. In the work of Larsson et al. precise information on evaporation conditions such as temperature and pressure are completely missing, rendering the interpretation of the results even more difficult.

Besides the removal of phenolics, we could also detect a clear decrease in acetol concentration by up to 84.5% at 200 mbar. This compound was included in the studies of Lange et al., who performed evaporation of volatile PAC components from the bioliq^®^ plant via heat treatment at 80 °C in open jars. They observed a decline in the acetol content by up to 77.7% compared to crude PAC after 1.5 h of treatment [12]. The slightly improved removal that was obtained in the recent work can be attributed to the reduction in pressure, which promotes evaporation. Even harsher conditions were applied in the work of Lian et al. since they performed their evaporation at the same temperature of 80 °C, but gradually decreased the pressure to values of up to 20 Torr (≈ 27 mbar). They stated to remove light volatile aldehydes and ketones like acetol, hydroxyacetaldehyde and furfural with this procedure, but unfortunately did not provide any analysis of their concentrations [10].

Considering our GC results, it becomes apparent that, at least under the harshest evaporation conditions, the contents of toxic components are all below or close to the growth limit of *A. oryzae*. Accordingly, growth on pure PAC should have been possible, but the fungal tolerance did not exceed 30%. This again indicates that the PAC must contain other substances that are toxic for fungal growth. In addition, it has already been shown in the literature that inhibitory components can also reinforce each other in their toxic effect [92–94].

Since rotary evaporation was the most effective single method tested in this work, but is quite time and energy consuming, a better alternative would be to use the acquired knowledge about the evaporation behavior of the individual PAC components for a targeted separation of the vapors already within the pyrolysis process. Such fractional condensations have already been described in the literature [95, 96] and could possibly provide fractions that can be utilized by the fungus even without extensive treatment.

#### Combination of detoxification methods and *A. oryzae* cultivation

A combination of the individual treatment methods further increased fungal tolerance and allowed growth on pure PAC. Various approaches combining several detoxification methods have been described in literature. For example, Hodge et al. used a combination of activated carbon and a subsequent overliming treatment with Ca(OH)_2_ on softwood hydrolysates. They found that the effective removal of phenolic compounds, which they obtained by treatment with 2.5–5% activated carbon, was not considerably enhanced by additional overliming [38]. This is consistent with the current findings, as the reduction in the concentration of the phenolic model compound guaiacol achieved by the AC treatment could not be improved by the subsequent overliming treatment, but remained constant at 0.14 g/L. In contrast, Hodge et al. were able to remove the furan HMF completely with the combination of AC + OL in almost all hydrolysates studied. We could confirm this observation, as we also noted a decrease in the concentration of furfural, i.e. our furanic model compound, from 0.4 g/L after single AC treatment to 0.05 g/L when combined with OL. Xu et al. evaluated the influence of the order of the two aforementioned detoxification methods on the concentrations of contained lignin, furfural and xylose sugars [36]. They determined activated carbon treatment following initial overliming to be the more effective chronological order, which coincides with our results. Even though PAC contained neither residual lignin nor sugar molecules, it was still tolerated to levels as high as 40% after OL + AC treatment, whereas the reverse order only resulted in a fungal tolerance of 10%. Based on the GC data, this observation can be mainly attributed to the better removal of ketones. Their concentrations were reduced by only 57.9% and 82.2% after treatment with AC + OL, while 82.8% and 91.2% less acetol and 2-cyclopenten-1-one were present in the PAC after OL + AC. This is confirmed by the study of Zhang et al. in which they give a comprehensive insight into the composition of a poplar prehydrolysate before and after detoxification with overliming and activated carbon via GC/MS and observed that the sequential treatment resulted in a removal of ketones [37]. A possible explanation for the improved detoxification with OL + AC was given by Randtke et al. as they reported the capacity of activated carbon to be improved by calcium, magnesium and sodium [97]. Since a large amount of NaOH is needed for overliming, this could have had a positive effect on the subsequent activated carbon treatment.

However, even for the combination of activated carbon and rotary evaporation, it seemed to be advantageous if the carbon treatment was performed at the end of the detoxification procedure. This was most likely due to the complete removal of 2-cyclopenten-1-one that could be achieved after RE + AC treatment, whereas the concentration of the highly toxic compound was still 0.24 g/L when the detoxification was conducted in the reverse order. Lian et al. also performed rotary evaporation combined with a subsequent activated carbon treatment to detoxify an aqueous phase formed during the pyrolysis of pelletized wood [10]. With their treatment, growth of *Cryptococcus curvatus* on pure condensate was enabled. In the current work, this could only be achieved by combining rotary evaporation, overliming and activated carbon treatment yielding a PAC that contained 2.96 g/L acetol and 0.14 g/L guaiacol as the only measurable inhibitors. However, the concentrations of both components were below the growth limits determined by Dörsam et al. [19], and it can therefore be assumed that these compounds did no longer cause any inhibition. It is even conceivable that the substances still remaining in the PAC could at least partly act as additional C-sources for the fungal growth. This assumption results from comparison of the acetate consumption and CDW formation obtained here with the data from the work of Kövilein et al. [42]. They performed a cultivation of *A. oryzae* on different acetate concentrations ranging from 5 to 70 g/L and observed a CDW of 1.14 g/L after 48 h in medium containing 40 g/L acetate, which is comparable to the content in the PAC. In the current work, a CDW of 3.0 g/L was formed within the same cultivation time, with the fungus consuming only 2.2 g/L acetate. The resulting high yield coefficient Y_X,S_ of 1.36 g/g indicates the utilization of alternative carbon sources. Further studies should therefore be conducted to determine whether the fungus is also capable of metabolizing other remaining PAC components such as acetol.

## Conclusions

In the current work, a comprehensive insight into the potential of several single detoxification methods as well as their combinations to increase the PAC tolerance of *A. oryzae* is provided. With an enzymatic treatment using *T. versicolor* laccase the content of phenolics could partly be reduced; however, the reduction did not result in any improvement in the fungal tolerance. The activated carbon treatment was also suitable to remove phenolics, but in addition the concentrations of acetol, 2-cyclopenten-1-one and furfural were decreased by 29.5%, 48.3% and 77.7%, respectively, when 10% AC was applied. This removal led to an increase in the fungal tolerance from 1.125 to 1.625% PAC.

The optimum conditions for an efficient overliming treatment were identified to be 100 °C and an initial pH of 12. Using these conditions and NaOH as alkaline agent, the growth limit of *A. oryzae* was raised to 12.5%. The GC analysis revealed that overliming is not well suited for the removal of phenolic compounds, but the concentrations of the main inhibitors 2-cyclopenten-1-one and furfural were remarkably reduced by 86.8% and 95.9%, respectively.

However, the most effective single method was identified to be rotary evaporation at 200 mbar, since with this treatment the concentration of every inhibitory compound analyzed was reduced to be below or at least close to the maximum tolerated by *A. oryzae,* leading to a fungal PAC tolerance of 30%. In the subsequent combination experiments, it became apparent that it is beneficial to perform the rotary evaporation in the beginning and the activated carbon treatment in the end of the detoxification procedure. Accordingly, the combination of RE + OL + AC was the only one to enable growth in 100% PAC shake-flask cultures leading to the formation of a maximum CDW of 5.21 ± 0.46 g/L. Consequently, the first step towards a microbial valorization of PAC with *A. oryzae* has been taken and future studies need to show whether the detoxification also enables a fungal malic acid production based on the pyrolysis condensate.

## Methods

### Microorganism and cultivation conditions

*Aspergillus oryzae* DSM 1863 was obtained from the DSMZ German Collection of Microorganisms and Cell Cultures (Deutsche Sammlung von Mikroorganismen und Zellkulturen GmbH, Braunschweig, Germany).

#### Preparation of the spore solution

For inoculation of the agar plates and shake flasks, glycerol stocks of the *A.* *oryzae* conidia were prepared. For this purpose, the fungus was cultivated using minimal medium according to Barratt et al. containing 10 g/L glucose, 6 g/L NaNO_3_, 0.52 g/L KCl, 0.52 g/L MgSO_4_·7H_2_O, and 1.52 g/L KH_2_PO_4_ [98], but with 2 mL/L 1000 × Hutner’s trace elements. The pH of the medium was adjusted to 6.5 with NaOH and 20 g/L agar were added before autoclaving for 20 min at 121 °C. The 1000 × Hutner’s Trace Element solution contains 5 g/L FeSO_4_·7H_2_O, 50 g/L EDTA-Na_2_, 22 g/L ZnSO_4_·7H_2_O, 11 g/L H_3_BO_3_, 5 g/L MnCl_2_·4H_2_O, 1.6 g/L CoCl_2_·6H_2_O, 1.6 g/L CuSO_4_·5H_2_O, and 1.1 g/L (NH_4_)_6_Mo_7_O_24_·4H_2_O, pH 6.5 [99]. The harvest of the spores was performed according to Kövilein et al. [42] and the final conidia concentration was set to 3 × 10^7^. Aliquots were prepared and stored at − 80 °C until use.

#### PAC tolerance tests on agar plates

The medium for the tolerance tests was similar to the one used for conidia preparation, but additionally defined volumes of PAC were added after autoclaving to avoid heat-induced compositional changes. The media were prepared as 2× concentrated stock solutions, which were diluted to their original concentration (1×) by adding the respective volumes of PAC and water. For inoculation 5 µL of spore solution with a final concentration of 1 × 10^7^ conidia per mL were dropped onto the middle of the plate. After 5 days of incubation at 30 °C the colony diameters were determined as the mean value of two measurements at an angle of 90°. All growth tests were performed as quadruplicates.

#### Shake-flask cultivation

The medium utilized for the shake-flask culture contained 4 g/L (NH_4_)_2_SO_4_, 0.75 g/L KH_2_PO_4_, 0.98 g/L K_2_HPO_4_, 0.1 g/L MgSO_4_·7H_2_O, 0.1 g/L CaCl_2_·2H_2_O, 5 mg/L NaCl, and 5 mg/L FeSO_4_·7H_2_O [100]. All medium ingredients were dissolved into detoxified PAC and 2 mL/L of 1000 × Hutners Trace Element solution were added.

The cultivation was performed at 100 mL scale in 500 mL baffled shake flasks containing 1 mL of 10% (v/v) Tween^®^ 80 solution to avoid fungal growth on the glassware. The cultures were inoculated by the addition of 1 mL spore solution (c = 3 × 10^7^ spores/mL) and incubated at 30 °C and 100 rpm for 96 h.

### Formation of the PAC

The PAC used in this work is derived from the fast pyrolysis of wheat straw, which is part of the bioliq^®^ process performed at KIT. A detailed description of the whole process was given by Pfitzer et al. [2]. Condensation of the vapors released during pyrolysis takes place in two successive stages, with the aqueous condensate being formed in the second condensation step at a temperature of 30 °C. The composition of the PAC was analyzed by the Thünen Institute of Wood Research in Hamburg and can be found in the appendix (Additional file 1: Table S1).

### Detoxification of the PAC

Prior to all treatment procedures (except the overliming), the pH of the PAC was adjusted to 6.5 using solid NaOH pellets. Afterwards the condensate was centrifuged at 4700×*g* for 10 min at room temperature and the supernatant was filtered through a paper filter (Macherey–Nagel, type MN615, cellulose) to obtain the PAC that was used as starting material for the detoxification experiments and that is henceforth referred to as the untreated control. The centrifugation conditions described in this section were used for all other centrifugation steps performed during the PAC treatment experiments.

#### Laccase treatment

The enzymatic treatment of the pyrolysis condensate was performed using *T. versicolor* laccase (38429, Sigma Aldrich) with a specific activity of > 0.5 U/mg. To determine the optimum enzyme amount and duration for PAC detoxification enzyme concentrations of 0; 1; 2.5; 5; 10 and 25 U/mL were added to 5 mL of the PAC and the mixtures were incubated for 24 h at 30 °C and 180 rpm. Samples of 200 µL were taken after 0 h, 0.5 h, 1 h, 2 h, 4 h, 8 h and 24 h and diluted 1:10 in 10% acetic acid immediately to perform a pH induced halt in the enzymatic reaction [44]. The experiments were performed in duplicates. For the growth test, the PAC was treated with 25 U/mL for 24 h.

#### Activated carbon

For the activated carbon treatment, carbon loads of 0%; 1.25%; 2.5%; 5% and 10% (w/v) were added to 100 mL PAC and the suspension was constantly stirred for 1 h at room temperature. To track changes in the PAC composition 5 mL samples were taken every 15 min and centrifuged immediately. The supernatant was then filtered through a 0.2 µm syringe filter and stored at 4 °C until analysis. After the incubation, the remaining mixture was centrifuged and residual carbon particles were removed by two successive vacuum filtration steps. The first step was conducted using a Büchner funnel and two layers of paper filters (Whatman, type 595) followed by a 0.2 µm bottle-top filtration (Thermo Scientific, PES). The experiment was performed in duplicates, but both batches were combined after the filtration and used for the *A. oryzae* growth test.

#### Overliming treatment

In general, the overliming procedure involved the pH increase of the completely unprocessed PAC (pH 2–3) to the alkaline range using either NaOH or Ca(OH)_2_. After pH adjustment, the condensate was stirred in a closed vessel in an oil bath at different temperatures. Afterwards, it was centrifuged and filtered through a paper filter (Macherey–Nagel, type MN615, cellulose). In case the pH value of the filtrate was still in the alkaline range, it was set to 6.5 using 96% H_2_SO_4_. To evaluate the influence of the temperature on the detoxification efficiency different oil bath temperatures ranging from 20 to 100 °C were tested. For this experiment, the initial pH values were set to 10. The alkaline PAC was incubated for 4 h and a sampling of 3 mL was conducted at hourly intervals. The experiment was performed in duplicates at 100 mL scale. For the pH experiments, the temperature was set to 80 °C and the incubation time was shortened to 90 min. Two alternative pH strategies were tested comprising the increase of the initial pH to 12 and the pH regulation at pH 10 throughout the overliming procedure. The experiment was performed at 500 mL scale and 15 mL samples were taken every 15 min. The pH of the regulated batch was manually set to a value of 10 again after every sampling using the same hydroxide that was applied for the initial pH increase.

#### Rotary evaporation

For rotary evaporation treatment, 500 mL of the condensate were poured into a 1 L round bottom flask and the oil bath temperature of the rotary evaporator was set to 80 °C. At the beginning of the procedure, the PAC was preheated for 10 min in the oil bath at atmospheric pressure and a rotation speed of 90 rpm. Following the preheating phase, the pressure was decreased gradually to values of 400, 300 and 200 mbar and the pressure was held for 4 h. After the evaporation, the condensate was refilled with ultrapure water to the initial volume to avoid a concentration of residual inhibitors. The experiments were performed in duplicates.

#### Combination of methods

In the combination experiment, the activated carbon treatment was performed using a carbon load of 10% (w/v) for 10 min. The overliming was carried out at 100 °C and an initial pH of 12 using NaOH pellets as alkaline agent. For the rotary evaporation, a pressure of 200 mbar was held for 4 h at the temperature and rotary speed already mentioned above.

### Analytics

#### Quantification of the fungal cell dry weight

For determination of the fungal cell dry weight the culture broth of a whole shake flask was filtered through pre-weighed paper filters. After thorough washing with ultrapure water, the biomass containing filters were dried in an oven at 70 °C until a constant weight was reached. The weight of the filters was measured using a precision scale and the CDW was expressed in g/L after subtraction of the filter blank weight.

#### Enzymatic quantification of acetate

The concentration of acetate contained in the pyrolytic condensate was analyzed using an enzyme assay (10148261035, R-Biopharm AG, Darmstadt, Germany). The final volume specified in the manual was scaled down either to a fourth or a twentieth when the quantification was performed in cuvettes or microtiter plates, respectively. In the cuvette scale the measurement was conducted according to the manufacturer’s instructions whereas in microtiter plates the concentration was determined using an acetate calibration curve in the range of 0.03–0.15 g/L.

#### Ammonium assay

The consumption of the nitrogen source during the *A. oryzae* shake-flask cultures was determined using the Spectroquant^®^ ammonium test kit (114752, Merck KGaA, Darmstadt, Germany). The measurement was performed following the procedure recommended by the manufacturer, except for the adaptation to microtiter plates by scaling down the volume to 200 µL.

#### Folin–Ciocalteu assay

The concentration of the total phenolic compounds in the PAC was quantified via microscale Folin–Ciocalteu colorimetry [101]. The procedure was scaled down to 1.5-mL cuvettes by halving the reaction volume. Hence, 10 µL of sample was added to a mixture of 0.79 mL of ultrapure water and 50 µL FC reagent. After vortexing the mixture was incubated for 5 min and 150 µL of 25% (w/v) sodium carbonate were added into the cuvettes. The solution was thoroughly mixed by vortexing again and incubated for 1 h at room temperature. The measurement was performed at 765 nm using a spectrophotometer (Spectronic 200, Thermo Scientific) and the amount of total phenolics was determined as gallic acid equivalents via a gallic acid calibration curve ranging from 0.05 to 1 g/L. Like the laccase samples, the single calibration points were prepared as 1:10 dilutions in 10% acetic acid to compensate for possible side reactions of the acetic acid with the FC reagent.

#### GC analysis of inhibitory compounds

The quantification of the inhibitory PAC compounds acetol, furfural, 2-cyclopenten-1-one, guaiacol and phenol was conducted using a GC system (Agilent 6850 series, Agilent, Germany) that was equipped with a DB WAX column (30 m × 0.25 mm ID × 0.25 μm film thickness) and an FID detector. A sample volume of 1 µL entered the system via a split–splitless injector, operated at a split ratio of 25:1 and 250 °C. Helium was used as carrier gas. The GC oven program started with a temperature hold at 40 °C for 1 min, followed by three 5 °C/min ramps to 60 °C, 120°, 200 °C, respectively. The temperature at the end of every ramp was held for 2 min. The GC was operated at a constant pressure of 0.489 bar. The concentrations of acetol and 2-cyclopenten-1-one were quantified via calibration curves ranging from 0.005 to 5 g/L. For the quantification of furfural, phenol and guaiacol the curves covered a range of 0.001–1 g/L.

#### HPLC analysis of acetate in *A. oryzae* shake-flask culture

To determine the acetic acid concentrations in the liquid cultures HPLC (Agilent 1100 Series, Agilent, Germany) measurements were performed using a Rezex ROA organic acid H + (8%) column (300 × 7.8 mm, 8 µm particle size; Phenomenex) and a Rezex ROA organic acid H + (8%) guard column (50 × 7.8 mm). Prior to the analysis the samples were centrifuged at 4700×*g* for 10 min to spin down the biomass and 10 µL of the supernatant was injected into the HPLC after appropriate dilution. The system was operated under isocratic conditions using 5 M H_2_SO_4_ as mobile phase at a flow rate of 0.5 mL/min. The oven temperature was kept at 50 °C and sample detection was achieved using a refractive index detector. Quantification was performed via an acetate calibration curve ranging from 0.1 to 5 g/L.

#### Statistical analysis

OriginPro Software [version 2020 (9.7)] was used for statistical analysis. First, normality of the data was assessed by Shapiro–Wilk tests. One-way and two-way ANOVA were then performed using Tukey as post hoc test at a significance level of *p* < 0.05.

## Supplementary Information

**Additional file 1: Table S1.** GC/MS analysis of the raw PAC according to Thünen Institute of Wood Research in Hamburg.

**Additional file 2: Fig. S1.** Temporal change of the pH value during 4 h overliming treatment at different temperatures using NaOH and Ca(OH)2. The data are mean values of duplicates and the error bars indicate the standard deviation.

## Data Availability

The datasets used and analyzed during the current study are available from the corresponding author on reasonable request.
