# Supplementary material for: Detoxification of a pyrolytic aqueous condensate from wheat straw for utilization as substrate in Aspergillus oryzae DSM 1863 cultivations
Source: Biotechnol Biofuels Bioprod. 2022 Feb 17;15:18. doi: 10.1186/s13068-022-02115-z (PMC8855548; doi:10.1186/s13068-022-02115-z)
Supplement: Supplementary file 2 — Additional file 2: Fig. S1. Temporal change of the pH value during 4 h overliming treatment at different temperatures using NaOH and Ca(OH)2. The data are mean values of duplicates and the error bars indicate the standard deviation. [file 13068_2022_2115_MOESM2_ESM.pdf]

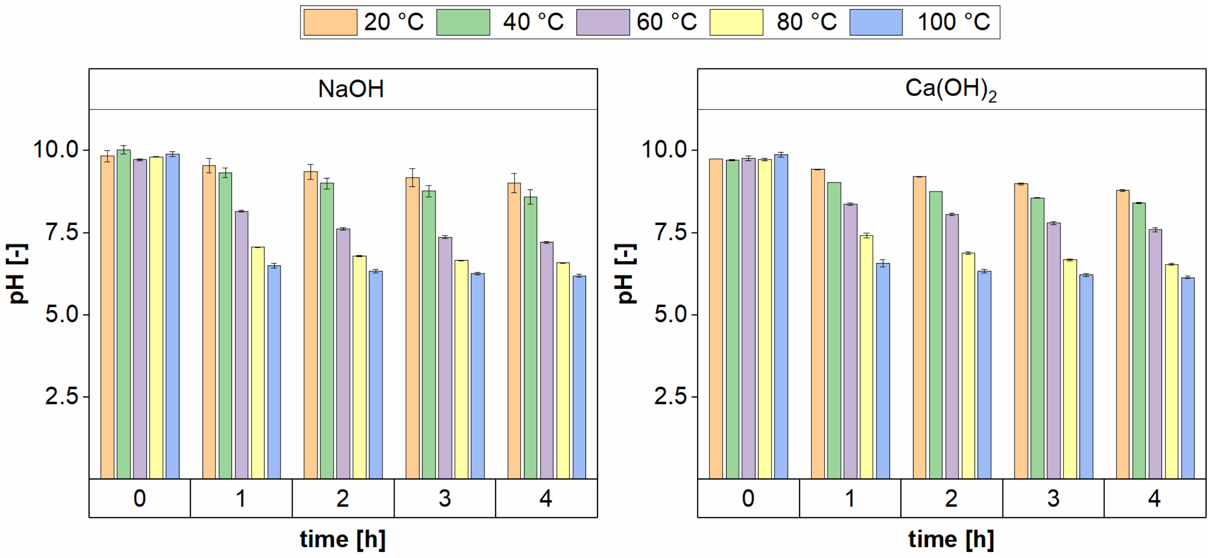

Fig. S1: Temporal change of the pH value during the 4 h overliming treatment at different temperatures using NaOH and $Ca(OH)_2$. The data are mean values of duplicates and the error bars indicate the standard deviation
